# Storage time and temperature affect the isolation rate of *Mannheimia haemolytica* and *Pasteurella multocida* from bovine bronchoalveolar lavage samples

**DOI:** 10.1186/s12917-020-02456-7

**Published:** 2020-07-13

**Authors:** Laura Van Driessche, Charlotte De Neve, Freddy Haesebrouck, Katharina van Leenen, Filip Boyen, Bart Pardon

**Affiliations:** 1 Department of Large Animal Internal Medicine, Faculty of Veterinary Medicine, Ghent University, Salisburylaan 133, 9820 Merelbeke, Belgium; 2 Department of Pathology, Bacteriology and Avian Diseases, Faculty of Veterinary Medicine, Ghent University, Salisburylaan 133, 9820 Merelbeke, Belgium

**Keywords:** *Pasteurellaceae*, Transport conditions, Bronchoalveolar lavage, Cattle, Bovine respiratory disease

## Abstract

**Background:**

A microbiological diagnosis is essential to better target antimicrobial treatment, control and prevention of respiratory tract infections in cattle. Under field conditions, non-endoscopic broncho-alveolar lavage (nBAL) samples are increasingly collected. To what extent the highly variable turnaround time and storage temperatures between sampling and cultivation affect the isolation rate of bacterial pathogens is unknown. Therefore, the objective of this experimental study was to determine the effect of different storage temperatures (0 °C, 8 °C, 23 °C and 36 °C) and times (0,2,4,6,8,24,48 h) on the isolation rate and concentration of *Pasteurellaceae* in nBAL samples from clinically affected animals.

**Results:**

At a storage temperature temperature of 36 °C isolation rates of *Mannheimia haemolytica* and *Pasteurella multocida* were significantly reduced 6 h and 48 h after sampling, respectively. At room temperature (23 °C), a decrease in *M. haemolytica* and *P. multocida* isolation rate was noticed, starting at 24 and 48 h after sampling, respectively, but only significant for *P. multocida* at 48 h. The presence of microbial contamination negatively affected the isolation of *P. multocida* in clinical nBAL samples, but not of *M. haemolytica*.

**Conclusion:**

Optimal *M. haemolytica* and *P. multocida* isolation rates from clinical nBAL samples are obtained after storage at 0 °C or 8 °C, provided that the sample is cultivated within 24 h after sampling. The maximum period a sample can be stored without an effect on the *M. haemolytica* and *P. multocida* isolation success varies and is dependent on the storage temperature and the degree of microbial contamination.

## Background

Respiratory tract infections (bovine respiratory disease (BRD)) have a major impact on farm economics and animal welfare [1]. Furthermore, they are the main indication for antimicrobial use in calves [2]. In order to rationalize antimicrobial use, diagnostic techniques need to be optimized. Non-endoscopic bronchoalveolar lavage (nBAL) is a practical and economical technique, increasingly used in Western European countries to sample the lower airways of cattle [3]. Although this technique obtains more pure cultures compared to a deep nasopharyngeal swab, microbial sample contamination can occur, partly depending on the experience of the veterinarian [3]. In order to minimize microbial contamination and bacterial overgrowth, cultivation of samples needs to be performed as soon as possible after sampling. However, due to the centralization of veterinary laboratories and the limited operating hours of these laboratories (not 24/7 as in some human clinics), the turnaround time between sampling and cultivating of the samples can take 24 h to even several days. Optimal storage conditions, supporting survival of causal pathogens and limiting growth of contaminants, are needed to obtain relevant bacterial analysis results [4]. False negative or irrelevant results may lead to therapy failure, resulting in increased antimicrobial use, antimicrobial resistance and mortality. Although storage conditions of clinical samples in the field are important, only few studies addressed this subject. Two studies are available comprising the effects of long term survival of *Pasteurellaceae*, namely in swabs from bears [5] or ovine and bovine tracheobronchial washings [6]. In the latter experiments, however, sterile lung fluids were spiked, and therefore possible contaminant effects were not taken into account. To what extent nBAL field samples can be stored until analysis, without influencing the isolation rate of clinically import pathogens, is currently unknown. Therefore, the objective of the present study was to determine the effect of storage temperature and duration on the isolation rate of *Pasteurellaceae* from bovine nBAL field samples.

## Results

Animals that met the inclusion criteria aged 1 week to 7 months. In total, 13 nBAL samples were eligable, of which the initial culture results at T0 showed 6 dominant cultures with 1 clinically relevant pathogen (4 *M. haemolytica*, 2 *P. multocida*) and 7 mixed cultures with both *M. haemolytica* and *P. multocida* (3 pure cultures, meaning the presence of 1 bacterial species on the agar plate with more than 2 colonies, containing only *M. haemolytica* and *P. multocida* and 4 dominant cultures with also some contaminants present). In total, *M. haemolytica* was isolated from 11 samples (84.6%) with an average log concentration of 4.0 at T0, and *P. multocida* from 9 samples (69.2%) with an average log concentration of 3.7 at T0. *Trueperella pyogenes* was isolated from one sample and *Moraxella bovis* from two samples at T0, both in mixed cultures. *H. somni* was not isolated. Contaminants present in the dominant cultures were *Streptococcus* spp., *Staphylococcus* spp., *Bacillus* spp., *Escherichia coli* and *Rothia nasimurium* with an average log concentration of 2.8 at T0.

**Fig. 1:**
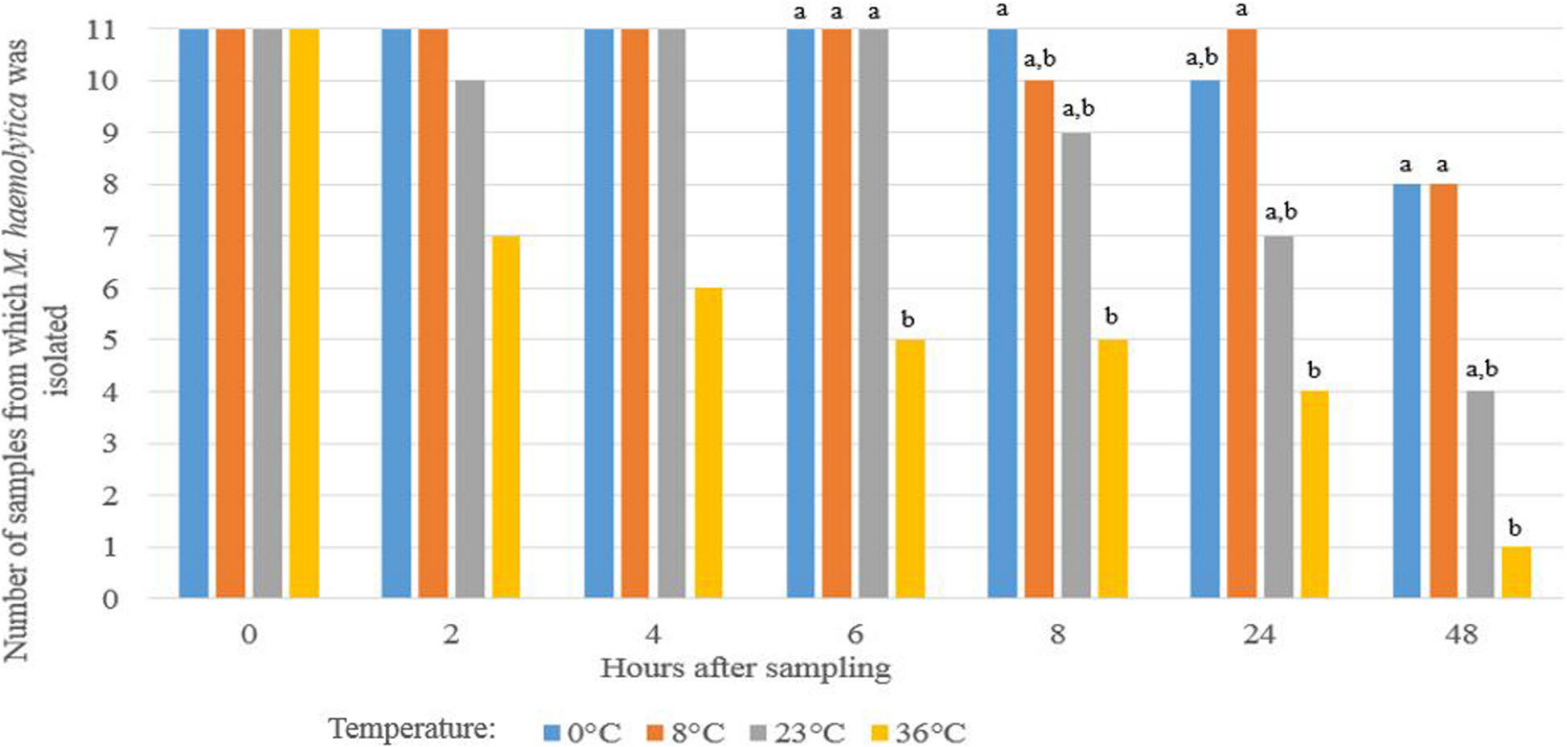
Effect of storage conditions (temperature and time) on the number of bovine nBAL samples from which *M. haemolytica* could be isolated. Different letters (a-b) indicate a significant difference (*P <* 0.05) in temperature within one time after sampling

The effect of the various temperatures and storage periods after sampling on the number of positive samples for *M. haemolytica* is presented in Fig. 1. In general, the number of samples from which *M. haemolytica* could be isolated, decreased gradually over time. The higher the storage temperature, the earlier the number of positive samples started to decrease, i.e. at 2 h of storage at 36 °C, at 24 h of storage at 23 °C and at 48 h of storage at 0 °C and 8 °C. During storage at 0 °C and 8 °C, the number of positive samples remained stable up to 24 h after sampling, varying between 10/11 and 11/11 in this period, whereas at 23 °C, only 7/11 samples remained positive after 24 h of storage. When samples were stored at 36 °C for 48 h, *M. haemolytica* could be isolated from only 1 sample, while this was 4/11 for 23 °C and 8/11 for both 0 °C and 8 °C (Fig. 1). Significance was only reached after 6 h of storage at 36 °C compared to lower temperatures.

**Fig. 2:**
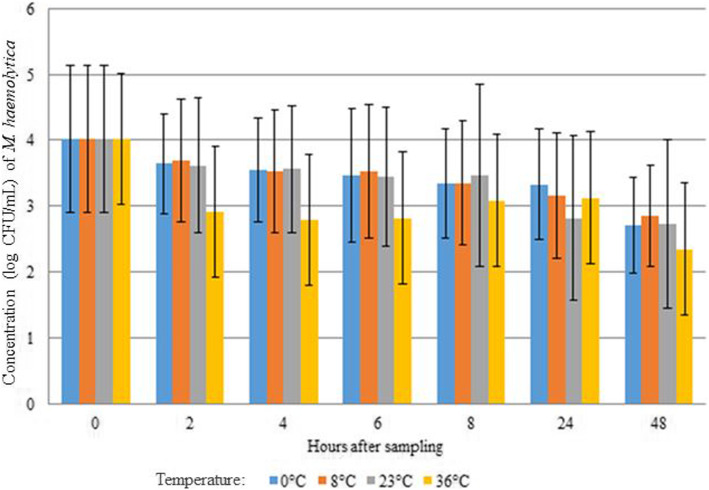
Effect of storage conditions (temperature and time) on the concentration of *M. haemolytica* in bovine nBAL samples. No statistically significant difference was seen between the different temperatures and the hours after sampling

An overall slight decrease in *M. haemolytica* concentration occurred over time (Fig. 2). At a storage temperature of 36 °C, the average log concentration of *M. haemolytica* decreased after 2 h of storage from 4 to 3.5 and remained stable until 48 h after sampling. No statistical significant difference was seen between the different temperatures and the time after sampling.

**Fig. 3:**
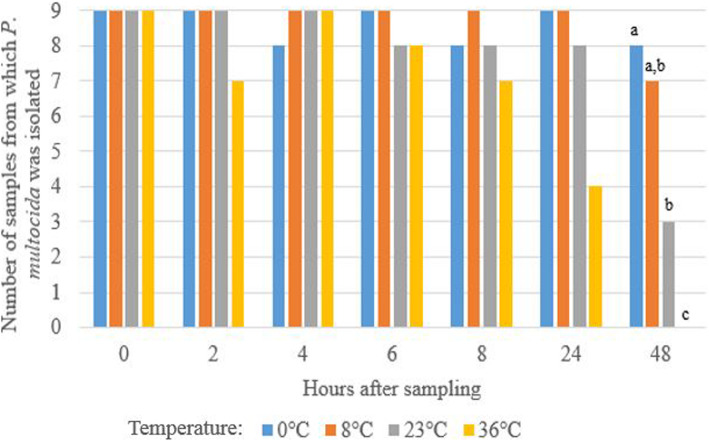
Effect of storage conditions (temperature and time) on the number of bovine nBAL samples from which *P. multocida* could be isolated. Different letters (a-c) indicate a significant difference (*P <* 0.05) in temperature within one time after sampling

The effect of storage temperature and time on the number of samples from which *P. multocida* could be isolated is presented in Fig. 3. A significant difference of isolation is only seen at 48 h of storage at a temperature of 0 °C or 8 °C, 23 °C and 36 °C. A decline in the number of positive samples from which *P. multocida* was isolated was observed throughout the experiment for storage at 36 °C, where only 4/9 positive samples were retrieved at 24 h of storage and no positive samples could be retrieved at 48 h (Fig. 3). This decline in the number of positive samples was due to both contaminant overgrowth and a decreased viability. This decreased viability of *P. multocida* was noticed at a storage temperature of 36 °C starting from 24 h after sampling and at a storage temperature of 23 °C starting from 48 h after sampling. When samples were maintained for 48 h at 23 °C, only 3/9 samples were found positive. At a storage temperature of 0 °C or 8 °C, the number of positive samples remained stable until 24 h after sampling, with an isolation rate of 8/9 to 9/9. At 48 h of storage at a temperature of 0 °C, 1 initially *P. multocida* positive sample was negative due to decreased viability. When stored at 8 °C for 48 h, 2 initially *P. multocida* positive samples were negative, one due to decreased viability and one due to contaminant overgrowth.

**Fig. 4:**
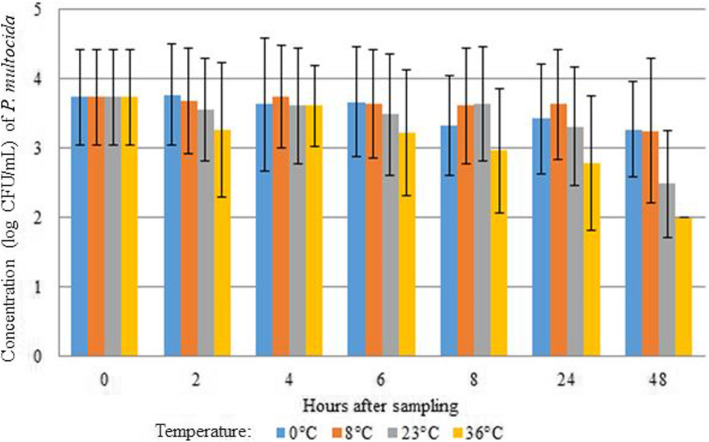
Effect of storage conditions (temperature and time) on the concentration of *P. multocida* in bovine nBAL samples. No statistically significant difference was seen between the different temperatures and the hours after sampling

Compared with *M. haemolytica*, the average concentration of *P. multocida* remained more stable until 24 h of storage at a temperature of 0 °C, 8 °C and 23 °C (Fig. 4). At a storage temperature of 36 °C, the *P. multocida* concentration slightly decreased starting from 6 h after sampling, with a concentration below detection limit at 48 h after sampling (Fig. 4). Accordingly, no positive samples of *P. multocida* were retrieved after storage at 36 °C for 48 h, even in the absence of contamination overgrowth. No statistical significant difference was seen between the different temperatures and the time after sampling.

**Fig. 5:**
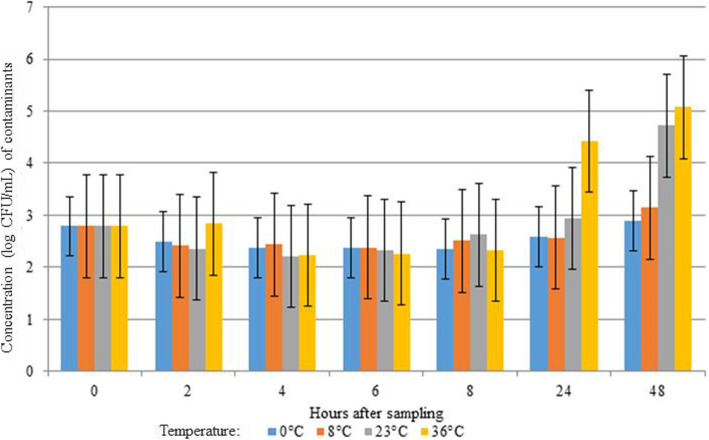
Effect of storage conditions (temperature and time) on the concentration of bacterial contaminants in bovine nBAL samples. No statistically significant difference was seen between the different temperatures and the hours after sampling

From the 13 samples collected, 10 samples contained microbial contamination at T0. Of the 3 initial samples that were not contaminated at T0, 1 sample showed microbial contamination starting from 2 h after sampling at a temperature of 36 °C. The other two initially negative samples showed sporadically microbial contamination with a concentration close to the detection limit. Results of the influence of bacterial contamination on the isolation rate of *M. haemolytica* and *P. multocida* in the clinical nBAL samples are presented in Table 1. A statistically significant negative association was seen between the presence of contaminants and the presence of *P. multocida*. An odds ratio of 0.32 was obtained for *P. multocida* (*P =* 0.04), meaning that the presence of contaminants reduced the odds of isolating *P. multocida*. In contrast, for *M. haemolytica* no significant effect of the presence of contaminants on isolation rates could be evidenced (*P =* 0.70). An average initial contaminants log concentration of 2.8 CFU/mL was observed (Fig. 5). This concentration remained stable during the first 8 h of storage, regardless of storage temperature. However, after 24 h of storage at 36 °C, the average contaminants log concentration increased to 4.4. After 48 h of storage, an average contaminants log concentration of 4.7 and 5.1 was reached for storage at 23 °C and 36 °C, respectively (Fig. 5).

**Table 1:** The effect of the presence of microbial contamination with a concentration of ≥1 × 10^2^ CFU/mL of each sample on the isolation of *M. haemolytica* and *P. multocida*

		Contaminants		Odds ratio	Confidence Interval	*P*-value
		Negative	Positive			
*Mannheimia haemolytica*	Negative	37.4%	62.2%	0.70	0.58–1.41	0.70
	Positive	45.8%	54.2%			
*Pasteurella multocida*	Negative	21.2%	78.8%	0.32	0.37–0.83	0.04
	Positive	45.7%	54.3%

## Discussion

This study describes the effects of different storage temperatures and times on the isolation rate and concentration of *Pasteurellaceae* from nBAL samples. When nBAL samples were stored at a temperature of 0 °C or 8 °C, high isolation rates of *P. multocida* and *M. haemolytica* were obtained until 24 h of storage. Since storage at 0 °C has low feasibility in routine practice, storage of clinical nBAL samples in a refrigerator is a readily available alternative for most practitioners. Also in previous studies low temperatures are recommended for storage [7, 8].

Even though there was no statistically significant decrease in the isolation rate after 24 h of storage at room temperature (23 °C) for both *P. multocida* and *M. haemolytica*, a probably relevant decrease in *M. haemolytica* isolation rate (from 11/11 to 7/11) was observed. The reasons for this decreased isolation rate were both a decreased viability (2/4 samples) and microbial contamination (2/4 samples). According to Tano et al. [9], clinically important bacteria can stay viable for 24 h at room temperature, but not in polymicrobial samples. However, these samples were spiked with high pathogen concentrations (10^6^ CFU/mL). When lower concentrations, comparable with concentrations obtained in the present study, were used (10^4^ CFU/mL and 10^5^ CFU/mL), results in viability varied. In another study *M. haemolytica* remained viable for a long period of up to 156 days [6]. However in that studies samples were also spiked with a high concentration (10^6^ – 10^7^ CFU/mL) and no bacterial contamination was present. These results stress the importance of a high initial pathogen concentration and avoiding microbial contamination during and after the sampling procedure, in order to maximize the probability to isolate *Pasteurellaceae*.

When samples were stored at 36 °C, the isolation rate started to decline already 2 and 24 h after sampling for *M. haemolytica* and *P. multocida*, respectively. When samples were maintained for 48 h at these temperatures, isolating clinically relevant pathogens was rare or no longer possible. This was mainly, but not exclusively, due to the increased concentration of contaminants, leading to uninterpretable samples when a concentration of ≥1 × 10^6^ CFU/mL of contaminants was reached. These results stress the importance of not leaving samples above room temperature, for example in a closed car or in a tropical environment.

The concentration of *M. haemolytica* slightly decreased over time independent of the storage temperature, though not statistically significant, while for *P. multocida*, this concentration remained more stable. Currently, no information is available on the survival rate of latter 2 bacteria in nBAL samples. One recent study describes the survival rate and density range of *Pasteurellaceae* in the nasopharyngeal microbiota in healthy calves [10]. This study showed a longer duration of carriage in the nose and higher concentration for *P. multocida* compared to *H. somni*, however rates of *M. haemolytica* were too low for meaningful survival modelling. Retaining high concentrations of relevant bacteria in clinical samples can be an added value to diagnostics, both for clinical interpretation as for direct detection methods using for example matrix-assisted laser desorption/ionization-time-of- flight mass spectrometry (MALDI-TOF MS) [11]. A higher negative association between the presence of contaminants and the isolation rate of *P. multocida* was found compared to *M. haemolytica*. This might be partially due to the fact that the average initial load of *M. haemolytica* in the samples was higher compared with the initial *P. multocida* load. Contaminant overgrowth might therefore negatively impact *P. multocida* isolation rate more quickly than the *M. haemolytica* isolation rate. A previous study showed that the growth of *M. haemolytica* can be inhibited by contaminants like *Escherichia coli* rather than *Staphylococcus* spp. or *Streptococcus* spp*.* [12]. To what extent different bacterial contaminants had an inhibitory effect on *P. multocida* and/or *M. haemolytica* in this study is unclear, considering the limited number of samples and since different bacterial contaminants were often combined at different concentrations within one clinical sample.

A limitation of the current study is the limited sample size. When using 8 positive samples per test group, only 60% difference in isolation rate could be detected. One of the reasons for the limited number of used samples are the strict inclusion and exclusion criteria applied. Indeed, only samples obtained from untreated calves, well-characterized as clinically affected at the level of the lower respiratory tract were included. In addition, only samples from which clinically relevant bacteria could be isolated at T0 were included in the experiment, resulting in the exclusion of various samples. Nevertheless, we feel that the current experimental set-up with a limited number of well-chosen samples and in-depth analysis of the obtained results allows drawing conclusions that are relevant for the practitioner. Another limitation of this study is that, considering the cultivation conditions used in this study, other relevant bacterial pathogens such as *Histophilus somni* and *Mycoplasma bovis* could not be isolated from the current clinical nBAL samples. However, *H. somni* is only rarely isolated because of poor viability and the fact that it is easily overgrown by other bacteria, either clinically relevant or not [3]. Although different studies are available describing the effect of storage conditions on the recovery of *M. bovis*, this was only investigated at low temperatures in milk samples [13, 14] or colostrum samples [15]. Therefore, further research into the effect of storage conditions on the recovery of *M. bovis* from nBAL samples is encouraged. Currently, the gold standard technique for identifying these fastidious bacteria is polymerase chain reaction (PCR). Since viability is not mandatory with this technique, it can be expected that the effect of storage conditions for identifying these pathogens will be less important in most veterinary labs using PCR to identify the latter pathogens. Moreover, maximizing the chance of isolating *P. multocida* and *M. haemolytica* is more critical since performing antimicrobial susceptibility testing in these species can be of major importance for appropriate antimicrobial treatment, while antimicrobial resistance is generally less prevalent in *H. somni* [16], although multiresistance has been described [17], or even not routinely tested for in *M. bovis.*

## Conclusion

This study demonstrates that optimal *M. haemolytica* and *P. multocida* isolation rates from clinical nBAL samples are obtained after storage at 0 °C or 8 °C, provided that the sample is cultivated within 24 h after sampling. The maximum period a sample can be stored without an effect on the *M. haemolytica* and *P. multocida* isolation success varies and is dependent on the storage temperature and the degree of microbial contamination.

## Methods

The sample size required to determine a 60% difference in isolation rate (80% vs 20%) with 80% power and 95% confidence for a 2-sided test was 8 positive nBAL samples per test group (Winepiscope 2.0, Zaragoza, Spain). Each test group comprises the presence of a clinically relevant bacterial pathogen, namely *Pasteurella multocida* and *Mannheimia haemolytica*. Therefore samples were taken until a minimum number of 8 positive culture results per test group was reached. An experimental study design was performed on 4 unrelated farms (3 beef, 1 dairy) between March and April 2018. Farms suffering from an acute outbreak of BRD were reported by local veterinarians and subsequently visited by the research staff. Animals to be sampled were selected based on previously described inclusion criteria [18]. Additionally, thoracic ultrasound examination was performed with a 7.5-MHz linear probe (Tringa Linear Vet, Esaote, the Netherlands) as previously described [19]. The definition for a case was the presence of a consolidated zone in the lung of ≥1 cm depth [20]. Animals that were treated with antimicrobials within 14 days prior to sampling were excluded from the study.

Cattle that met the inclusion criteria were sampled with the nBAL procedure as previously described [21]. Briefly, after disinfecting the nostril with 70% alcohol, a reusable home-made polytetrafluorethylene catheter adjusted with a 12-G catheter stylet was inserted in the nasal cavity and gently advanced, through larynx and trachea, into the bronchi. Next, 60 mL of sterile 0.9% NaCl was injected into the lungs and immediately aspirated (recovery of 30–50% of the fluid). Samples were transported at ambient temperature and further processed within 30 min after sampling.

Twenty mL of each nBAL sample was used for further analysis and was divided equally over four different 50 mL Falcon tubes after vortexing for 1 min (5 mL each). Each Falcon tube was incubated at a different temperature, all monitored with a thermometer, i.e. 0 °C +/− 1 °C (ice), 8 °C +/− 1 °C (refrigerator), 23 °C +/− 1 °C (room temperature) and 36 °C +/− 1 °C (incubator) for 0, 2, 4, 6, 8, 24 and 48 h. After each incubation period, the sample was vortexed for 30 s and 100 μL was transferred to an Eppendorf tube, already containing 900 μL phosphate buffered saline (PBS). Ten-fold dilutions were made of each sample for quantitative analysis as previously described [22]. From each dilution, 100 μl was inoculated on Columbia agar with 5% sheep blood (blood agar; Oxoid, Hampshire, UK) and incubated overnight at 35 °C +/− 2 °C in a 5% CO_2_ atmosphere. All macroscopically different colonies were counted and identified with MALDI-TOF MS as previously described [23]. A positive culture result was defined as the macroscopically visible presence of one or more clinically relevant *Pasteurellaceae* (*P. multocida* and *M. haemolytica*) colonies in pure, dominant or mixed cultures at T0 as previously described [3]. Contamination of these pure culture samples can occur over time due to incubation. Only samples with a positive culture at time point 0 h (T0) were included in the experiment. When no clinically relevant pathogen could be isolated at time points 2, 4, 6, 8, 24 or 48 h, the concentration of the pathogen isolated at T0 in this sample was set at 100 Colony forming units (CFU)/mL (being the detection limit of this plating procedure). A reduced viability of a certain bacterial pathogen was determined when the initial concentration declined linearly over time without an increase of the concentration of contaminants. An increased microbial contamination was determined as an increase of concentration of contaminants over time, until eventually the clinically relevant pathogen cannot be determined anymore.

The association between the different conditions for the isolation of *Pasteurellaceae* was determined by means of a multivariable logistic regression model with repeated measures (PROC GLIMMIX). Bonferroni corrections were used to compare between 4 groups. To determine the association between the presence of contaminants and isolation of *M. haemolytica* or *P. multocida* logistic regression was used (PROC LOGISTIC). Model validity was evaluated by the Hosmer-Lemeshow goodness-of-fit test for logistic models. Significance was set at *P* < 0.05. All data were collected in Microsoft Excel and statistical analysis was performed in SAS 9.4 (SAS Institute Inc., Cary, NY).

## Data Availability

The dataset used and analysed during the current study is available from the corresponding author on reasonable request.

## Abbreviations

BRD: Bovine respiratory disease
CFU: Colony forming unit
MALDI-TOF MS: Matrix-assisted laser desorption/ionization-time-of- flight mass spectrometry
nBAL: Non-endoscopic bronchoalveolar lavage
PCR: Polymerase chain reaction
